# Sepsis prediction during outbreaks at neonatal intensive care units through body surface screening for Gram-negative bacteria: systematic review and meta-analysis

**DOI:** 10.1186/s13104-018-4033-y

**Published:** 2018-12-22

**Authors:** Thomas Harder, Sebastian Haller, Tim Eckmanns, Juliane Seidel

**Affiliations:** Department for Infectious Disease Epidemiology, Robert Koch Institute (RKI), Seestraße 10, 13353 Berlin, Germany

**Keywords:** Systematic review, Meta-analysis, Outbreaks, Prognostic accuracy, Gram-negative bacteria

## Abstract

**Objective:**

This systematic review focusses on the prognostic accuracy of neonatal body surface screening during outbreaks caused by Gram-negative bacteria for prediction of sepsis. In a previous systematic review we reported that only limited evidence of very low quality exists regarding the predictive value of this screening under routine conditions. We aimed to investigate whether this is different in outbreak settings.

**Results:**

We identified five studies performed during outbreaks in three countries, comprising a total of 316 infants. All studies were at high risk of bias. In outbreak settings, pooled sensitivity of body surface screening to predict sepsis was 98% (95 CI 60 to 100%), while pooled specificity was 26% (95% CI 0.5 to 96%). Evidence quality was low for all outcomes. Extending a previously published systematic review, we show here that in contrast to routine settings sensitivity of body surface screening for sepsis prediction is very high, while specificity is still insufficient. Surface screening appears to be a useful component of bundles of interventions used during outbreaks, but the evidence base is still limited. PROSPERO Registration Number: CRD42016036664.

**Electronic supplementary material:**

The online version of this article (10.1186/s13104-018-4033-y) contains supplementary material, which is available to authorized users.

## Introduction

At neonatal intensive care units (NICUs), outbreaks caused by Gram-negative bacteria are an important public health problem. Management of such outbreaks includes the implementation of complex interventions, comprising isolation, hygiene measures and antimicrobial therapy. Body surface screening of newborns is often performed as part of this bundle of interventions [1]. However, the significance of screening within this bundle is unclear.

Recently, we published a systematic review showing that only limited evidence of very low quality exists regarding the sensitivity and specificity of these screening procedures for the prediction of sepsis in routine settings. Moreover, we observed that over all published studies, sensitivity was as low as 41%, while specificity was only 56% [2]. However, we did not include reports on outbreaks in this former systematic review, for the following reasons: Screening for colonization by Gram-negative bacteria is likely to perform differently during outbreaks of Gram-negative bacteria, compared to routine settings. During an outbreak, the increase of incidence influences positive as well as negative predictive values. Therefore, during the conduct of the project we decided to split the data base and to analyze outbreaks separately. In addition, we used this systematic review to address the issue of applying methods of evidence-based public health to outbreak reports as part of the piloting phase of the Project on a Framework for Rating Evidence in Public Health (PRECEPT) [3].

## Main text

### Methods

The systematic review reported here builds upon a systematic review for which the protocol has been published in the International Prospective Register for Systematic Reviews (PROSPERO; registration no. CRD42016036664). It was performed according to the Preferred Reporting Items for Systematic Reviews and Meta-analyses (PRISMA) statement (see Additional file 1: Table S1 for the completed checklist) [4]. For a detailed description of the methodology of the previous review, see [2]. In brief, electronic databases searched were MEDLINE and EMBASE. In addition, for the current review we additionally searched the Worldwide Database for Nosocomial Outbreaks (https://www.outbreak-database.com) for additional publications, using an adapted search string (date of last search: 29 June 2018). For complete search strategy, see Additional file 1: Table S2.

To be eligible, a study had to:

- Include infants up to an age of 12 months who are in a NICU (irrespective of gestational age and birth weight) AND.
- Report on an outbreak at a NICU caused by a Gram-negative bacteria species AND.
- Report the results of body surface screening for Gram-negative bacteria AND.
- Report on late-onset sepsis in these infants.

As in the previous review [2], we did not make any restrictions regarding study design, language or publication status (published/unpublished). From the eligible studies, two independent reviewers (TH, JS) extracted study characteristics and assessed risk of bias, using standardized forms. In case of disagreement, a final decision was made by consensus.

As in the previous review [2], for risk of bias assessment the QUADAS (Quality Assessment of Diagnostic Accuracy Studies)-2 tool [5] was used. Risk of bias was judged to be “high”, “low” or “unclear”. We used the methodology of the GRADE (Grading of Recommendations Assessment, Development and Evaluation) working group to assess the quality of the evidence for each body of evidence (true positives, true negatives, false positives and false negatives) [6].

As reported earlier [2], for quantitative data synthesis on prognostic accuracy, 2 × 2 tables were constructed to calculate sensitivity and specificity. Summary estimates using hierarchical summary receiver operating characteristics (HSROC) models and summary receiver operating characteristics (SROC) plots were constructed, accounting for the correlation between sensitivity and specificity [7].

### Results

A total of 3871 entries were identified in Medline and Embase. In addition, 227 potentially relevant outbreaks were identified in the Worldwide Database for Nosocomial Outbreaks. During the screening process, four studies [1, 8–10] were found to be eligible. One study [8] comprised two separate studies; therefore we finally included five studies into the analysis (see Additional file 2: Figure S1). The characteristics of these studies are shown in Table 1.

**Table 1:** Characteristics of included studies

Study	Country	Study period	No. of participants (in final analysis)	Gestational age (weeks)	Birth weight (g)	Age at screening (days)
Hill et al. 1974 (I) [8]	USA	1972	31	28–38	1100–3380	3–90
Hill et al. 1974 (II) [8]	USA	1972	23	28–38	1100–3380	3–90
Parry et al. 1980 [9]	USA	1978	128	NR	NR	NR
Samuelsson et al. 2014 [1]	Sweden	2006–2011 (recurrent outbreaks)	38	25 (36 for controls)	725 (2570 for controls)	NR
Tsiatsiou et al. 2015 [10]	Greece	2011	96	26–40	800–4300	10–80

Study	Screening interval (s)	Screening location	Outbreak bacteria species/strain	Definition of sepsis	Control measures	Risk of bias
Hill et al. 1974 (I) [8]	Weekly	Rectal/respiratory	*Klebsiella pneumoniae* type 26	NR	Enhanced handwashing; use of long-sleeved gowns; isolation	High
Hill et al. 1974 (II) [8]	Weekly	Rectal/respiratory	*Klebsiella pneumoniae* type 26	NR	Enhanced handwashing; use of long-sleeved gowns; isolation	High
Parry et al. 1980 [9]	Daily	Nose/throat/umbilicus/rectum	*Citrobacter diversus*	NR	Closure of nursery; sterile cleaning; cohorting	High
Samuelsson et al. 2014 [1]	Weekly	Nose/throat/perineum/rectum	*Serratia marcescens*	Positive blood culture plus ≥ 2 additional criteria; or: negative blood culture plus ≥ 3 additional criteria^a^	Hand hygiene; cleaning; handling of venous catheters; distribution of patients in room; antibiotics	High
Tsiatsiou et al. 2015 [10]	Weekly	Perianal/stool	Carbapenem-resistant *Acinetobacter baumannii*	NR	Antimicrobial therapy; closure of department to new admissions	High

*NR* not reported

^a^Additional criteria: (1) leucocyte particle conc. < 5 × 10^9^/L or > 20 × 10^9^/L; (2) platelet particle conc. < 100 10^9^/L; (3) C-reactive protein > 15 mg/L; (4) impaired respiratory function with respiratory rate > 70 breaths/min, grunting/gasping or increased ventilator support in ventilated infants that cannot be explained by other factors

The included studies were performed between 1972 and 2011 in three different countries and comprised a total of 316 infants. The outbreaks reported in the studies were caused by four different bacteria species (*Klebsiella pneumoniae*, *Citrobacter diversus*, *Serratia marcescens*, *Acinetobacter baumannii*). Birth weight ranged from 725 to 4300 g, while gestational age ranged from 25 to 40 weeks. None of the studies reported on ethnicity of participants. In all but one study, screening was performed once a week. Only one of the studies reported the definition used for sepsis. All five studies reported on control measures used to manage the respective outbreak.

The results of the risk of bias assessment using the QUADAS-2 tool are summarized in the last column of Table 1. As confounding of the predictive performance of the screening due to co-interventions (measures applied to control the outbreak) cannot be excluded in any of the studies, all five studies were judged to be at high risk of bias.

Table 2 shows sensitivity, specificity, prevalence, positive and negative predictive values for the five included studies. All but one study had 100% sensitivity in detecting the respective pathogen. This corresponded to high negative predictive values in the respective studies. Specificity was high (81–93%) in three studies, but zero in two. Consequently, positive predictive values ranged between 18 and 57%.

**Table 2:** Measures of prognostic accuracy of included studies

Study	Sensitivity (%)	Specificity (%)	Prevalence of sepsis (%)	Positive predictive value for sepsis (%)	Negative predictive value for sepsis (%)
Hill et al. 1974 (I) [8]	92	0	39	37	0
Hill et al. 1974 (II) [8]	100	81	9	33	100
Parry et al. 1980 [9]	100	93	2	18	100
Samuelsson et al. 2014 [1]	100	0	26	26	–^a^
Tsiatsiou et al. 2015 [10]	100	93	8	57	100

^a^Cannot be calculated

**Fig. 1:**
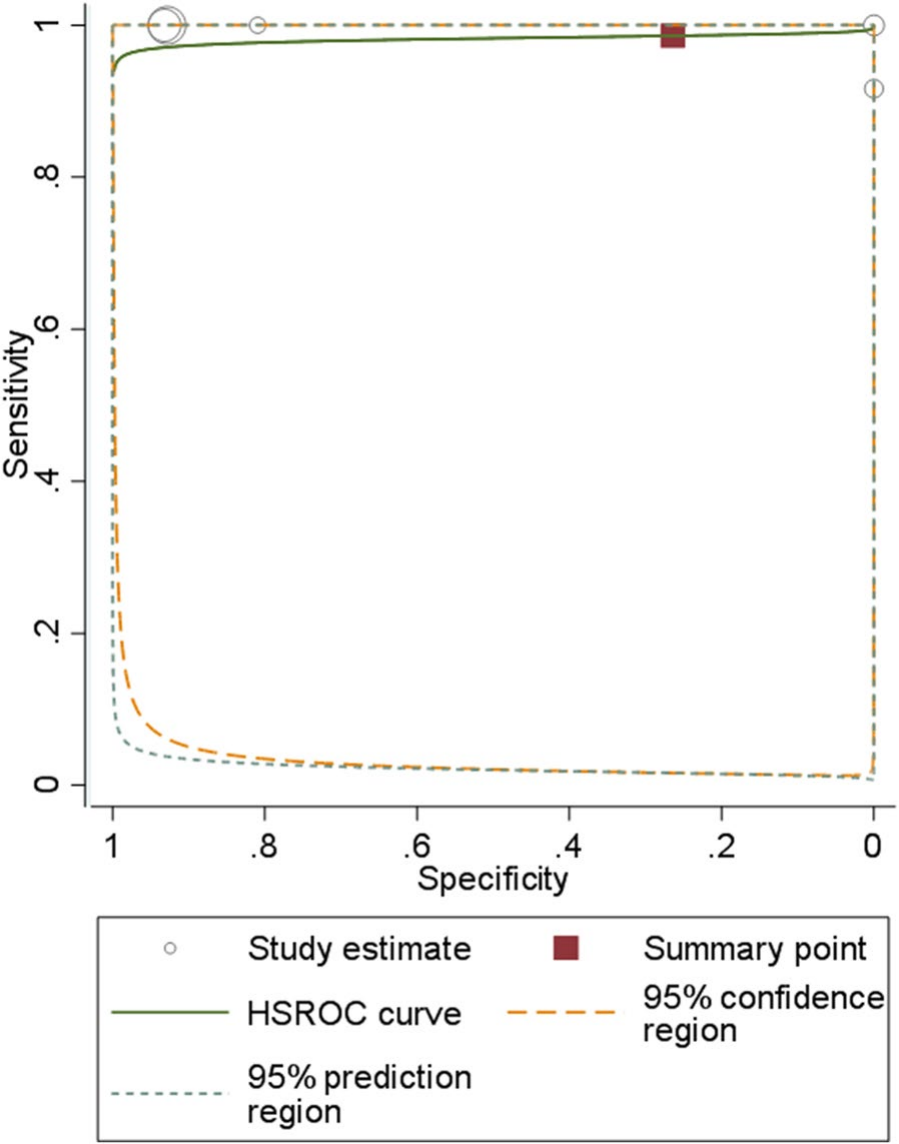
Summary receiver operator characteristics (SROC) curve

Although the results of the single studies showed heterogeneity, we decided to pool sensitivity and specificity measures to get overall estimates. Pooled sensitivity across all studies was 98% (95% CI 60% to 100%), while pooled specificity was 26% (95% CI 0.5% to 96%). Pooled diagnostic odds ratio was 25.2 (95% CI 0.04 to 14542). Figure 1 shows the summary receiver operating characteristics (SROC) plot.

According to GRADE, evidence quality for all four outcomes (true positives, true negatives, false positives, false negatives) was assessed to be low. This was due to high risk of bias and inconsistency of study estimates.

### Discussion

In this study, we extended a previously published systematic review on the performance of body surface screening in neonates at NICUs to outbreaks settings. In contrast to routine settings where both sensitivity and specificity of the screening were found to be low [2], during outbreaks sensitivity of sepsis prediction by colonization by Gram-negative bacteria was nearly 100%, whereas specificity was still insufficient. Low specificity may be explained by the fact that carriers of Gram negative outbreak pathogens are predominantly colonized and do not necessarily develop infection. Infection rates depend on factors of the pathogen (e.g. virulence), the host (e.g. immunodeficiency) and the route of transmission (e.g. transmission during invasive procedure versus by skin contact) and do differ from outbreak to outbreak. The low specificity of the screening is of relevance when considering it for clinical routine, where screening results may be misinterpreted as strong predictors of Gram-negative sepsis. Consequently, this may result in less prudent antibiotic administration.

In outbreak situations it is of importance to identify all cases in order to apply adequate control measures and understand the mode of transmission. Beyond identification of infants at risk for developing sepsis, the major objective of screening is to identify all infants carrying the outbreak pathogen. The here found low specificity in predicting sepsis should not hinder from performing a systematic screening in outbreak situations to implement hygiene measures.

In any case one has to consider that the evidence base comprised only report of five outbreaks, and evidence quality was low due to high risk of bias.

This extension of a previously published systematic review has several strengths. Using a structured approach and an established evidence grading system, we were able to conduct the first systematic review on this topic in outbreak situations. By focusing on outbreaks, we investigated a setting where the background prevalence of both colonization and disease (sepsis) can be expected to be considerably higher than under routine conditions.

## Limitations

The limitations of this systematic review are mainly caused by the limited evidence base. In the previous review on routine screening, we were able to perform subgroup analyses according to sampling site and bacteria species. Interestingly, these analyses revealed that, under routine conditions, the screening performed differently in some bacteria species. Unfortunately, such an analysis was not possible here as the evidence base was too small. As in the previous review, risk of bias clearly limits the value of the data and decreases the quality of the evidence.

The main result of our systematic review confirms that during outbreaks caused by Gram-negative bacteria, body surface screening in neonates has a high sensitivity in detecting newborns at risk of sepsis. Thereby, the screening procedure can be considered to be a useful component of a bundle of interventions used during outbreaks and is prerequisite for the control of outbreaks that are caused by person to person transmission. However, data are more heterogeneous regarding specificity of the screening. Ideally, more studies should be conducted to broaden the evidence base.

## Additional files

**Additional file 1: Table S1.** PRIMA checklist. **Table S2.** Search strategy for Medline and Embase and search strategy for worldwide database for nosocomial outbreaks.

**Additional file 2: Figure S1.** Flow chart.

## Abbreviations

GRADE: grading of recommendations assessment, development and evaluation
HSROC: hierarchical summary receiver operating characteristics
NICU: neonatal intensive care unit
PRECEPT: Project on a Framework for Rating Evidence in Public Health
PRISMA: Preferred Reporting Items for Systematic Reviews and Meta-analyses
PROSPERO: International Prospective Register for Systematic Reviews
QUADAS: Quality Assessment of Diagnostic Accuracy Studies
SROC: summary receiver operating characteristics
