# Supplementary figures and images for: Sepsis prediction during outbreaks at neonatal intensive care units through body surface screening for Gram-negative bacteria: systematic review and meta-analysis
Source: BMC Res Notes. 2018 Dec 22;11:917. doi: 10.1186/s13104-018-4033-y (PMC6303936; doi:10.1186/s13104-018-4033-y)

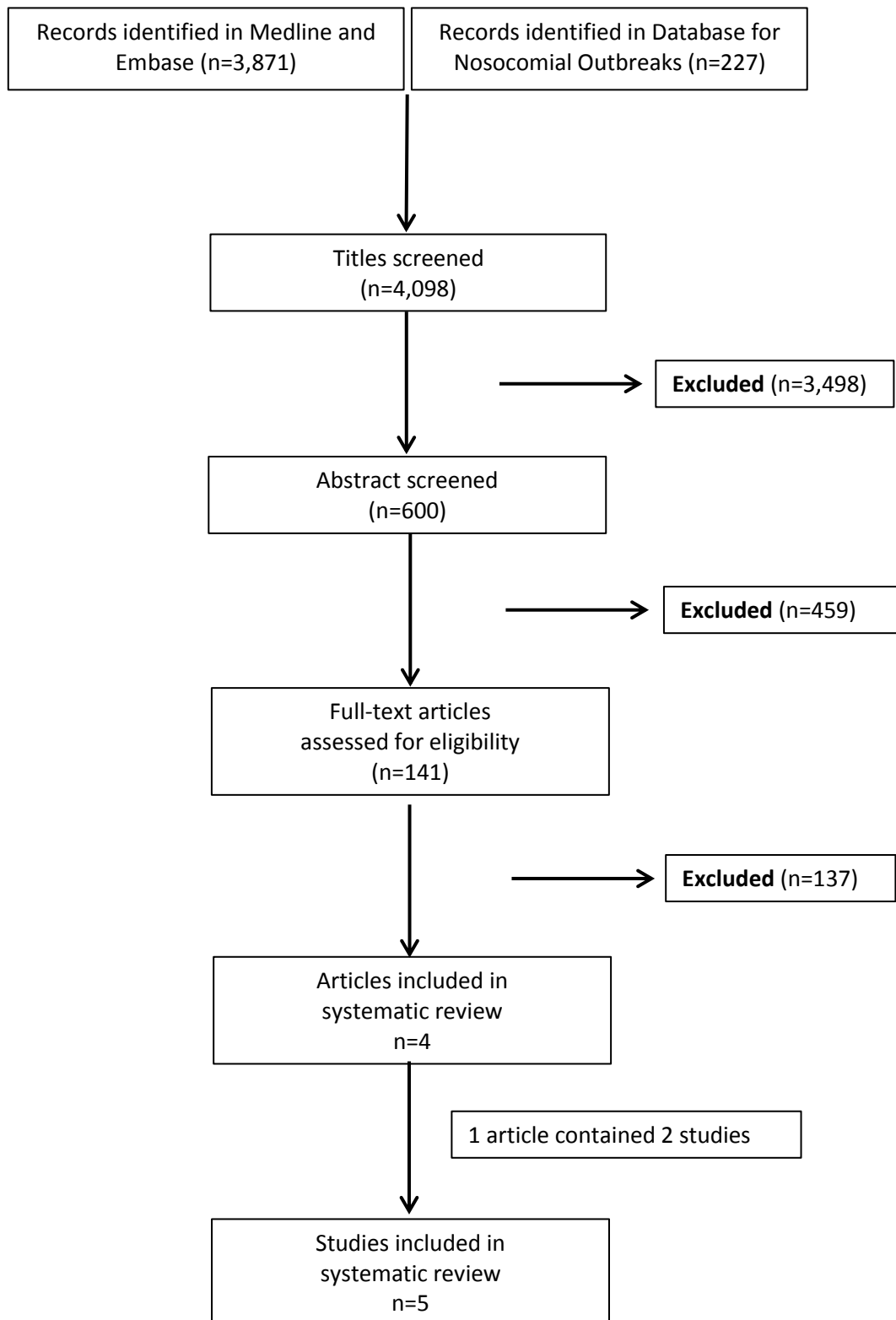

Supplement: Supplementary file 2 — Additional file 2: Figure S1. Flow chart. [file 13104_2018_4033_MOESM2_ESM.pdf]
